# Supplementary material for: Hyperbranched Polymers Modified with Dansyl Units and Their Cu(II) Complexes. Bioactivity Studies
Source: Materials (Basel). 2020 Oct 14;13(20):4574. doi: 10.3390/ma13204574 (PMC7602284; doi:10.3390/ma13204574)
Supplement: Supplementary file 1 [file materials-13-04574-s001.pdf]

materials

MDPI

Article

Hyperbranched polymers modified with dansyl units and their Cu(II) complexes. Bioactivity studies

Paula Bosch [1], Desislava Staneva [2], Evgenia Vasileva-Tonkova [3], Petar Grozdanov [3], Ivanka Nikolova [3], Rositsa Kukeva [4], Radostina Stoyanova [4] and Ivo Grabchev [5]

[1] Institute of Science and Technology of Polymers, ICTP-CSIC, Juan de la Cierva 3, 28006 Madrid, Spain; pbosch@ictp.csic.es
[2] Department of textile and leathers, University of Chemical Technology and Metallurgy, 1756 Sofia, Bulgaria; grabcheva@mail.bg
[3] The Stephan Angeloff Institute of Microbiology, Bulgarian Academy of Sciences, 1113 Sofia, Bulgaria; evaston@yahoo.com (E.V.-T.); grozdanov_bg@yahoo.com (P.G.); vanianik@mail.bg (I.N.)
[4] Institute of General and Inorganic Chemistry, Bulgarian Academy of Sciences, 1113 Sofia, Bulgaria; rositsakukeva@yahoo.com (R.K.); radstoy@svr.igic.bas.bg (R.S.)
[5] Faculty of Medicine, Sofia University "St. Kliment Ohridski", 1407 Sofia, Bulgaria
* Correspondence: i.grabchev@chem.uni-sofia.bg

1.1. Spectral Analysis and Characterisation

Absorption spectra were taken on a "Thermo Spectronic Unicam UV 500" spectrophotometer, while the fluorescence ones - on a "Cary Eclipse" spectrophotometer. All organic solvents were of spectroscopic grade and used without special treatment. The spectra were recorded using 1 cm path length quartz glass cells. Absorption and fluorescence measurements were carried out at a 10^{-5} mol l^{-1} concentration. FT-IR spectroscopic analyses of cotton fabrics were performed using an IRAffinity-1 spectrophotometer (Shimadzu Co., Kyoto, Japan) equipped with a MIRacleTM ATR. 2.4. Automatic analyser EA 3000 (Euro Vector, Italy) with accuracy of analysis: ± 0, 01% absolute error has been used for elemental analysis.

1.2. Preparation of the Cotton Fabric for SEM

The cotton fabrics—virgin and treated with polymers S1 and S2 and their Cu(II) complexes - were incubated overnight for 24 h in meat-peptone broth (MPB) inoculated with *B. cereus* cell suspension. After the incubation, the samples were washed, dried and coated with gold, and then investigated on a Jeol JSM-5510 scanning electron microscope.

1.3. EPR analysis

EPR spectra of the complexes were recorded as the first derivative of the absorption signal using a Bruker EMXplus EPR spectrometer, operating in the X-band (9.4 GHz). The recording temperature was varied within the 120-450 K range. The quantitative EPR calculations were performed by SpinCountTM software module (Bruker). The spectra were simulated by the SIMFONIA program (Bruker).

1.4. Antimicrobial Tests

The antimicrobial activity of the polymers samples S1 and S2 and their Cu(II) complexes was determined in vitro by the agar diffusion method and broth dilution shaking. The following model strains were used (Institute of Microbiology collection, Sofia, Bulgaria): Gram-positive bacteria *Bacillus cereus* ATCC 11778, Gram-negative bacteria *Pseudomonas aeruginosa* 1390 and the yeasts *Candida lipolytica* 7618. In the agar diffusion method, Mueller-Hinton agar (MHA) plates were seeded with aliquots of cell suspensions of the cell cultures. The tests were performed using 0.4% solutions of the investigated compounds in DMSO, of which equal amount (40 μL) was added into wells (8

mm in diameter) punched in MHA. Commercial discs with gentamicin (G) and nystatin (Ns) were used as a standard antibacterial and antifungal agent, respectively. The plates were incubated at appropriate temperature and monitored for growth for 24-48 h. The antimicrobial activity was indicated by the presence of clear zones around the wells (mm in diameter, including well/disc).

1.5. Minimum Inhibitory Concentration (MIC)

Broth dilution test was used for MICs determination of the investigated compounds against the test cultures [1]. Serial dilutions of the compounds were prepared in test tubes with meat-peptone broth (MPB) in the range of 20–400 μg/mL. Control tubes without added compounds were also prepared for each microbial culture. The tubes were inoculated with respective microbial suspension and incubated at appropriate temperature for 24h. The microbial growth was determined by the turbidity of the medium at 600 nm (OD_{600}). The growth control, sterility control and sample control were used. The lowest concentration of the samples that inhibited the visible growth of the strains was referred as MIC. Three independent experiments were carried out and averages were taken.

1.6.. Antibacterial Activity of Modified Cotton Fabrics

Cotton fabrics treated with the compounds were tested for antibacterial activity against *B. cereus* and *P. aeruginosa* as model strains. Test tubes containing MPB and square cotton specimens (10 mm x 10 mm) were inoculated with suspension of each bacterial culture. Tubes with untreated cotton and without specimen were also prepared as controls. After incubation for 24 hat appropriate temperature, the specimens were removed and OD_{600} was determined. The antimicrobial activity of the treated cotton samples was evaluated by the reduction of OD_{600} after incubation compared to the control sample. All antimicrobial tests were done in triplicate and the average was taken.

1.7. Cellular toxicity

HEp-2 cells (National Bank for Industrial Microorganisms and Cell Cultures, No. NBIMCC-95, Sofia, Bulgaria) were grown in medium containing 10% heated calf serum in DMEM (Gibco BRL, USA) supplemented with 10 mmol/L HEPES buffer (Gibco BRL, USA) and antibiotics (penicillin, 100 U/mL; streptomycin, 100 μg/mL).

Monolayer cells in 96-well plates (Costar®, Corning Incorporated, Kennebunk, USA) were inoculated with 0.1 mL/well containing concentrations (in logarithmic intervals) of the compounds diluted in a maintenance medium. The cells were incubated in a humidified atmosphere at 37°C and 5% CO_2 for 48 h. After microscopic evaluation, the maintenance medium containing the test compound was removed, the cells were washed and 0.1 mL maintenance medium supplemented with 0.005% of neutral red dye was added to each well and the cells were incubated at 37°C for 3 h. After incubation, the neutral red day was removed, and the cells were washed once with phosphate buffered saline (PBS) and 0.15 mL/ of well desorb solution (1% glacial acetic acid and 49% ethanol in distilled water) was added. The optical density of each well was read at 540 nm (OD_{540}) in a microplate reader (Organon Teknika Reader 530). The 50% cytotoxic concentration(CC_{50}) was defined as the material concentration that reduced the cell viability by 50% when compared to untreated control.

References

1. Wikler, MA. *Methods for Dilution Antimicrobial Susceptibility Tests for Bacteria That Grow Aerobically: Approved Standard.* 5th ed. Wayne, PA: National Committee for Clinical Laboratory Standards (NCCLS); 2000, M7–M5.

© 2020 by the authors. Submitted for possible open access publication under the terms and conditions of the Creative Commons Attribution (CC BY) license (http://creativecommons.org/licenses/by/4.0/).
